# Extensive subcutaneous emphysema of the thigh as a rare complication following total knee arthroplasty: A case report

**DOI:** 10.1016/j.ijscr.2023.108466

**Published:** 2023-07-07

**Authors:** Kenji Takami, Shigeyoshi Tsuji

**Affiliations:** a Department of Orthopaedic Surgery, Nippon Life Hospital, Osaka, Japan; b Department of Rehabilitation, Nippon Life Hospital, Osaka, Japan

**Keywords:** Emphysema, Complication, Total knee arthroplasty, Case report

## Abstract

**Introduction and importance of the case:**

This is the first report of subcutaneous emphysema of the thigh as a complication after total knee arthroplasty (TKA).

**Presentation of case:**

A 78-year-old female patient with valgus knee arthropathy underwent TKA. Two days postoperatively, the patient experienced left thigh swelling and pain, and subcutaneous emphysema was detected upon palpation. Although the skin tone was comparable to the other side, the left thigh was tender and firm. The surgical wound did not exhibit erythema. Computed tomography imaging revealed emphysema in the subcutaneous and intermuscular regions of the left thigh. Gram stain and culture tests from arthrocentesis were negative, and blood culture results were also negative. As there was no fever or disturbance of consciousness, and the LRINEC score was 1, supportive care was provided to the patient. At 5 days postoperatively, there was an observable improvement in the emphysema, and by day 9 postoperatively, the emphysema had fully resolved.

**Clinical discussion:**

There is a lack of documented cases reporting extensive subcutaneous emphysema of the thigh following TKA, suggesting it to be an exceedingly rare complication. In this case, we conducted a thorough investigation to assess the potential association of infection. Subsequently, the symptoms were successfully alleviated with supportive care without antibiotics.

**Conclusion:**

The occurrence of subcutaneous emphysema in the thigh was identified as a postoperative complication following TKA. Blood tests, culture tests and LRINEC score can be valuable tools for differentiation.

## Highlights

• The complication after total knee arthroplasty involves thigh subcutaneous emphysema.
• The challenge of subcutaneous emphysema is its distinction from infections.
• Blood tests, culture tests and LRINEC score can be valuable tools for differentiation.

## 1 Introduction

Total knee arthroplasty (TKA) has established itself as a means of relieving pain and restoring knee joint function [[1], [2], [3]].

Although TKA is generally considered a safe procedure for relieving pain and restoring knee joint function, certain complications, such as infection and deep venous thrombosis, can occur [1,4].

In addition to the above, while postoperative subcutaneous emphysema is a known complication of total hip arthroplasty and open reduction internal fixation, there have been no reported cases of this complication following TKA [5,6].

This report documents a case of femoral subcutaneous emphysema occurring after a TKA procedure.

the work has been reported in line with the SCARE criteria [7].

## 2 Case description

**Fig. 1:**
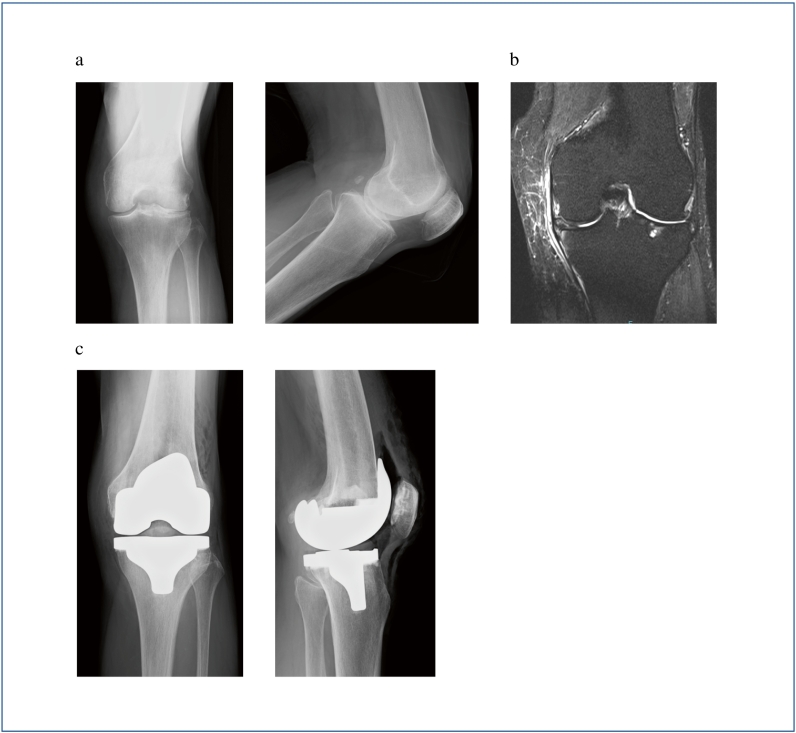
Preoperative and postoperative radiographs and preoperative MRI. a) The preoperative radiographs showed osteoarthritis (OA) findings in both compartments, although they were predominantly in the lateral compartment. b) The MRI was also taken before the surgery. c) Postoperative radiographs. The surgery was successfully completed without any complications during the operation.

**Fig. 2:**
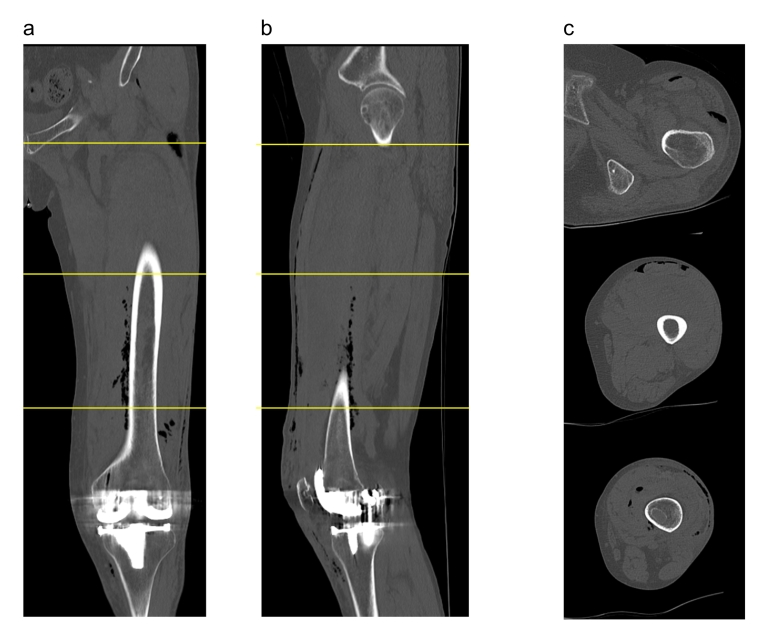
A CT scan was performed on the second day after the operation. a) The coronal image reveals the presence of emphysema extending to the hip joint. b) The sagittal image displays emphysema along the quadriceps fascia and around the femur. c) Three axial images, from proximal to distal, demonstrate extensive spread of emphysema.

**Fig. 3:**
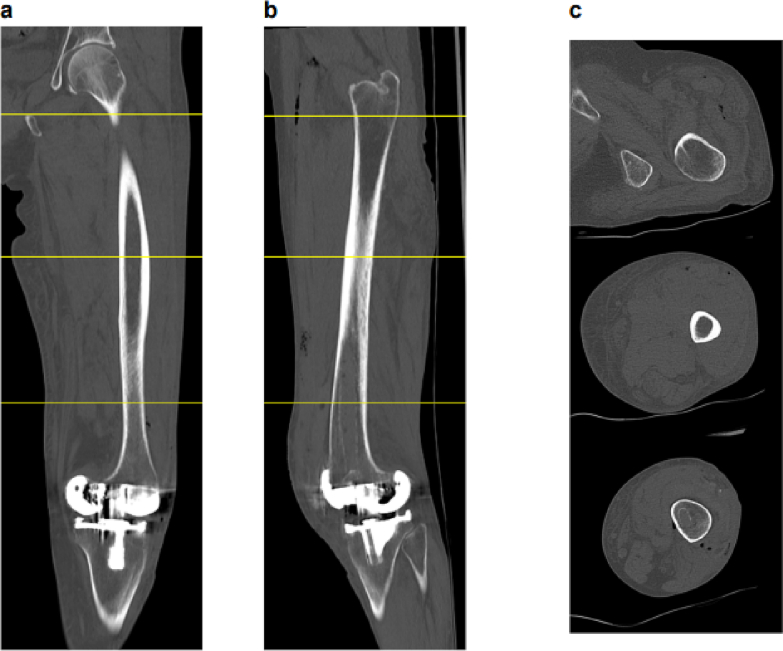
A CT scan was performed on the fifth day after the operation. a–c) Overall, the emphysema is resolving.

**Fig. 4:**
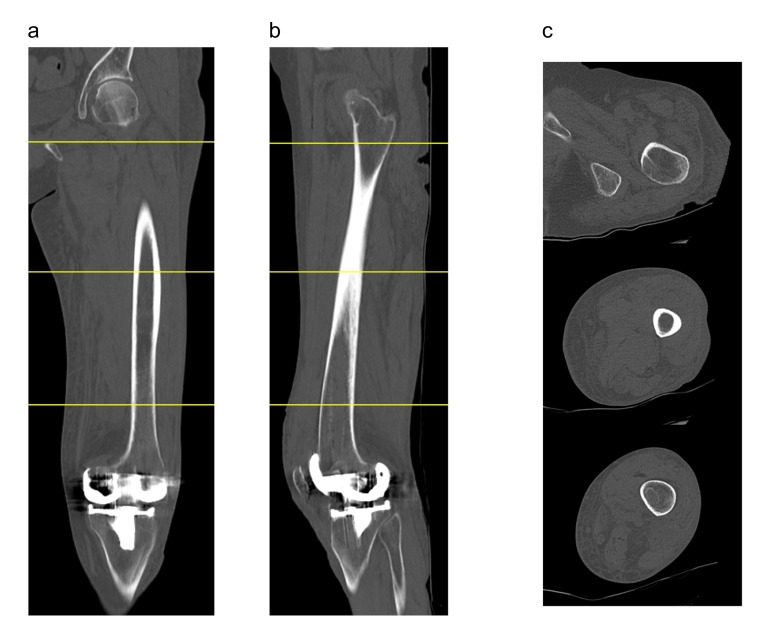
A CT scan was performed on the ninth day after the operation. a–c) The emphysema resolved.

A 78-year-old female patient was referred to our clinic due to left knee pain that had persisted for 3 months. The affected knee joint exhibited a slightly limited range of motion with 10 degrees of flexion-contraction and 140 degrees of flexion, compared to 5 degrees of flexion-contraction and 140 degrees of flexion on the contralateral side. The patient's continuous walking distance was limited to about 200 m due to pain. The knee joint was diagnosed with Kellgren-Laurence grade III arthropathy with a valgus deformity, although this type of arthropathy is relatively rare in Japan. The patient had a history of asthma and had been on inhaled steroid medication since the age of 30. Additionally, she had hypertension, dyslipidemia, and diabetes mellitus, which was controlled with one DPP4 inhibitor, with preoperative HbA1c of 6.3 % and fasting blood glucose of 110 mg/dL. At age 64, she had undergone an arthroscopic meniscectomy due to an anterior to posterior horizontal tear of the lateral meniscus, which was considered the likely reason for her osteoarthritis with lateral compartment dominance. TKA was performed using a lateral parapatellar approach, which was completed without complications (Fig. 1). However, two days after surgery, the patient reported left thigh swelling and pain, with palpable subcutaneous emphysema. The left thigh was firm and tender, although there was no difference in skin tone compared to the contralateral side. The surgical wound was not erythematous but continued to bleed slightly. Computed tomography imaging revealed emphysema in the subcutaneous and intermuscular areas of the left thigh (Fig. 2). Although infection with gas-producing bacteria was a potential differential diagnosis and extensive thigh emphysema after TKA had never been reported, Gram stain and culture were negative according to arthrocentesis. Similarly, the blood culture was negative. Since there were no signs of fever or disturbance of consciousness, and the LRINEC score was 1, the patient was managed with supportive care without antibiotics. Blood tests showed that the inflammatory response peaked 4 days after surgery (Table 1, Table 2). Both procalcitonin and presepsin remained negative during daily examination from postoperative day 3 to 5 (Table 1). At 5 days postoperatively, the emphysema showed a trend toward improvement (Fig. 3), and by 9 days postoperatively, the emphysema had resolved (Fig. 4). At the 6-month follow-up after surgery, no signs of infection were observed at the surgical wound site. Although the range of motion was restricted to 5 degrees of flexion-contraction and 110 degrees of flexion, the patient was able to ambulate without pain and without an assistive device.

**Table 1:** Laboratory data after surgery.

Days after surgery		1	3	4	5	8
AST	U/L	20	13	14	14	14
ALT	U/L	9	6	6	6	6
LDH	U/L	181	147	137	149	186
T-BIL	mg/dL	0.9	1.1	1	1.1	1.6
D-BIL	mg/dL	0.2	0.3	0.3	0.3	0.5
ALP	U/L	50	58	56	68	63
γ-GTP	U/L	22	22	28	32	29
TP	g/dL	6.6	6.2	5.8	5.8	5.9
Albumin	g/dL	3.8	3.3	3	3	3.1
CK	U/L	134	45	27	29	28
Amylase	U/L	118	51	44	48	51
BUN	mg/dL	23	29.2	31.6	31.2	26.1
Creatinin	mg/dL	0.72	0.84	0.82	0.83	0.76
eGFR	ml/min/1.73 m^3	58.8	49.7	51	50.3	55.4
Uric acid	mg/dL	6.3	6.4	6.6	6.5	5.7
Na	mmol/L	140	138	139	137	136
K	mmol/L	4.3	4.5	3.9	4.2	3.8
Cl	mmol/L	104	101	101	100	99
Ca	mg/dL	9	9	8.5	8.6	8.8
IP	mg/dL	3.6	2.6	2.8	3	2.7
Mg	mg/dL	1.7	2.1	2.1	2.1	2.1
CRP	mg/dL	2.26	11.66	12.55	8.98	5.89
D-dimer	μg/ml	2	4.2	6.6	NA	NA
Procalcitonin	ng/ml	NA	0.1	0.09	0.08	NA
Presepsin	pg/ml	NA	370	371	364	NA
WBC	*10^3/μL	12.52	10.04	7.17	6.03	6.96
NEUT	%	85.5	78.3	71	71.4	80
LYMP	%	7.6	13.1	19.2	18.4	11.5
MONO	%	6.7	7.7	7.5	8.3	7.8
EOS	%	0	0.7	2	1.7	0.3
BASO	%	0.2	0.2	0.3	0.2	0.4
RBC	*10^6/μL	4.42	3.62	3.31	3.23	3.11
Hemoglobin	g/dL	13.8	11.3	10.4	10.2	9.4
Hematocrit	%	42.1	34.7	31.6	31	29.7
PLT	*10^3/μL	135	119	126	165	200

AST: aspartate aminotransferase; ALT: alanine aminotransferase; LDH: lactate dehydrogenase; T-BIL: total bilirubin; D-BIL: direct bilirubin; ALP: alkaline phosphatase; γ-GTP: gamma-glutamyltranspeptidase; TP: total protein; CK: creatine kinase; BUN: blood urea nitrogen; eGFR: estimated glomerular filtration rate; Na: sodium; K: potassium; Cl: chlorine; Ca: calcium; IP: inorganic phosphorus; Mg: magnesium; CRP: C-reactive protein; WBC: white blood cell count; NEUT: neutrophil; LYMP: lymphocyte; MONO: monocyte; EOS: eosinophil; BASO: basophil; RBC: red blood cell count; PLT: platelet; NA: not available.

**Table 2:** Fasting blood glucose data after surgery.

Days after surgery	1	2	3	4	5	6	7	8
Fasting blood glucose (mg/dL)								
Before breakfast	134	130	135	107	110	126	110	139
Before lunch	174	158	171	126	117	135	129	138
Before dinner	167	160	122	125	136	147	107	134
Before sleep	153	156	149	146	140	145	136	157

## 3 Discussion

TKA is a common treatment for knee osteoarthritis and is currently performed at a rate of 100,000 per year in the UK and 700,000 per year in the US [3]. TKA has a long history of use, and the results have been improving due to advancements in surgical techniques and models [[8], [9], [10]]. While the risk of complications has decreased accordingly, certain complications such as infection and deep vein thrombosis are still inevitable [1,4]. The frequency of infection varies among reports but is approximately 1 % in registries from New Zealand, Australia, and the US. Risk factors include increasing age, obesity, alcohol abuse, diabetes, smoking, malnutrition, urinary tract infection, liver disease, an American Society of Anesthesiologists scale >2, systemic and local corticosteroid therapy, blood transfusion, and immunosuppression, among others [[11], [12], [13]]. No previous reports have documented postoperative development of subcutaneous emphysema, as observed in this case. Although there are scattered reports of subcutaneous emphysema as a postoperative complication of total hip arthroplasty or open reduction internal fixation [5,6], this is the first report of subcutaneous emphysema of the thigh after TKA. In previous reports, low body mass index, reaming, mechanical traction [6], and reoperation [14] were proposed as potential causes, although none were definitive. In the present case, the patient had undergone reoperation and mechanical traction, and had bronchial asthma, using inhaled steroids for nearly 50 years, which may have contributed to soft tissue fragility [15,16].

Typically, the occurrence of thigh swelling, firmness, and pain, accompanied by palpable subcutaneous emphysema, is not observed following TKA, leading us to recognize an aberration in the patient's condition. Given the gravity of the situation, our primary concern was to prioritize the exclusion of gas-producing bacterial infection as a crucial step, considering its urgent requirement for surgical intervention. Clinical findings, blood tests such as C-reactive protein, procalcitonin, and presepsin [17], and culture tests are helpful in distinguishing infection. In addition, the LRINEC score is particularly useful when necrotizing soft tissue infection is suspected [18,19]. In the blood tests, the levels of creatinine, sodium, C-reactive protein, white blood cell count, and fasting blood glucose were within normal ranges, each receiving a score of 0. The hemoglobin level was 11.3, warranting a score of 1. Consequently, the calculated LRINEC score was determined to be 1. In the present case, arthrocentesis was performed, and joint fluid Gram stain and culture examination were negative. Similarly, blood culture was negative. Since the patient did not present with fever or disturbance of consciousness, and the LRINEC score was 1, there were no findings suggestive of infection. The emphysema improved spontaneously without the use of antimicrobial agents with supportive care, and the patient's activities of daily living gradually improved.

## 4 Conclusion

In summary, this is the first report of subcutaneous emphysema of the thigh as a complication after TKA. This condition is challenging to differentiate from infection and requires careful clinical judgment. However, blood tests, culture tests and LRINEC score can be valuable tools for differentiation.

This case report was reported based on the latest SCARE guidelines [20].

## Ethical approval

The case report was approved by the Ethics Review Committee of Nippon Life Hospital on 3rd May 2023. (approval number 2023-011).

## Funding

None.

## Author contribution

KT drafted the manuscript. ST approved the final manuscript.

## Guarantor

Kenji Takami has accepted full responsibility for this work and the decision to publish it.

## Research registration number

Not applicable.

## Consent

Written informed consent was obtained from the patient for the publication of this case report and accompanying images. A copy of the written consent form is available for review by the Editor-in-Chief of this journal on request.

## Provenance and peer review

Not commissioned, externally peer-reviewed.

## Declaration of competing interest

None.
